# The Effect of Nanocoatings Enriched with Essential Oils on ‘Rocha’ Pear Long Storage

**DOI:** 10.3390/foods9020240

**Published:** 2020-02-24

**Authors:** Custódia Gago, Rui Antão, Cristino Dores, Adriana Guerreiro, Maria Graça Miguel, Maria Leonor Faleiro, Ana Cristina Figueiredo, Maria Dulce Antunes

**Affiliations:** 1 MED, FCT, Universidade do Algarve, Campus de Gambelas, 8005-139 Faro, Portugal; cgago@ualg.pt (C.G.); a39270@ualg.pt (R.A.); cristino.dores@gmail.com (C.D.); mgmiguel@ualg.pt (M.G.M.); 2 CEOT, FCT, Universidade do Algarve, Campus de Gambelas, 8005-139 Faro, Portugal; acguerreiro@ualg.pt; 3 CBMR, FCT, Universidade do Algarve, Campus de Gambelas, 8005-139 Faro, Portugal; mfaleiro@ualg.pt; 4 CESAM Lisboa, CBV, DBV, Faculdade de Ciências da Universidade de Lisboa, Campo Grande, 1749-016 Lisboa, Portugal; acfigueiredo@fc.ul.pt

**Keywords:** superficial scald, internal disorders, *Pyrus communis*, *Cymbopogon citratus*, citral

## Abstract

The effect of coating ‘Rocha’ pears with alginate-based nanoemulsions enriched with lemongrass essential oil (LG) or citral (Cit) was investigated. Fruit were treated with the nanoemulsions: sodium alginate 2% (*w*/*w*) + citral 1% (*w*/*w*) (Cit1%); sodium alginate 2% (*w*/*w*) + citral 2% (*w*/*w*) (Cit2%); sodium alginate 2% (*w*/*w*) + lemongrass 1.25% (*w*/*w*) (LG1.25%); sodium alginate 2% (*w*/*w*) + lemongrass 2.5% (*w*/*w*) (LG2.5%). Then, fruit were stored at 0 °C and at 95% relative humidity, for six months. Fruit samples were taken after two, four and six months, and then placed at 22 °C. Upon removal and after 7 d shelf-life, fruit were evaluated for colour CIE (L*, h◦), firmness, soluble solids content (SSC), titratable acidity (TA), weight loss, electrolytic leakage, microbial growth, symptoms of superficial scald and internal browning. All nanoemulsions had droplets in the nano range <500 nm, showed uniformity of particle size and stable dispersion. Cit-nanoemulsions had lower droplet size and higher stability than LG. No nanoemulsion showed cytotoxicity. Coatings reduced fruit colour evolution and preserved better firmness than control. After shelf-life, better firmness was found in LG-coated fruit. Coatings did not affect SSC and TA. Microbial growth was below the safety limits in all treatments. Fruit treated with LG-nanoemulsions did not show scald symptoms and panelists preferred LG1.25% coated fruit. Cit2% treated fruit showed the highest scald and internal browning symptoms, while LG1.25% did not show any disorders. This study suggests that LG-nanocoatings have the potential for preserving the quality of ‘Rocha’ pear.

## 1. Introduction

‘Rocha’ pear is an important crop with long storage potential, being stored for up to 10 months under a controlled atmosphere, nevertheless, they can develop a chilling injury in long term cold storage [1].

Superficial scald is a chilling-induced oxidative disorder developed in pear fruit after prolonged storage at −0.5 to 0.0 °C, which can be prevented by antioxidants [2,3] but they are no longer allowed in the European Union. After this interdiction, 1-methylcyclopropene (1-MCP) was introduced as an alternative to those antioxidants being effective on reducing scald [2], but, not without causing some problems in the normal ripening of the ‘Rocha’ pear [3].

Recently, several technologies, such as edible coatings containing antimicrobials and antioxidants, have been used for the preservation of fruit [4,5,6]. The antioxidant properties of those edible coatings can be an alternative to improve quality and extend the storage life of fruit commodities by delaying the metabolic processes [4,5,6] and they may be an effective auxiliary to control scald development.

For the formulation of edible coatings, special attention has been given to the essential oils (EOs) as functional biocompounds, which can be used as natural flavourings and antimicrobials in food [7]. EOs are complex mixtures of natural volatile compounds that can be isolated from aromatic plants [8]. Their lipophilic nature facilitates its interactions with microbial membranes causing their disruption [7,8]. Although the benefits of EOs in food matrices are recognized as antioxidants and antimicrobials, their low water solubility, intense aroma or potential toxicity at high concentrations are matters of concern [9]. Consequently, the search for systems able to slowly release bioactive compounds into food matrices is a challenge in food technology [8].

The leaves of lemongrass (*Cymbopogon citratus* (DC.) Stapf), when crushed, deliver a characteristic pleasant aroma, which has allowed the use of this plant and/or their EOs in perfumery and food industries [10]. Citral, a mix of two isomers (geranial and neral), predominates in the lemongrass oil, which seems to have an important role in the antimicrobial activity reported for lemongrass EOs. This antimicrobial activity has led to the use of them for preserving foods, particularly for their activity against diverse bacteria and fungi [11].

Alginate is a common polysaccharide largely used in emulsion formulations applied as edible coatings in many fruits, such as apple and strawberries [5,12,13].

Recently, nanoemulsions oil in water (O/W) were considered colloidal dispersions of oil droplets with particle size diameters lower than 500 nm [14]. Additionally, nanoemulsions incorporating essential oil have been successfully used for microbial control on fruit commodities [15]. Nevertheless, their use in fruit safety and preservation through long-term storage still needs clarification.

The objectives of this study were to develop alginate-based nanoemulsions supplemented with EOs, suitable for coating ‘Rocha’ pears, preserving or even improving their quality during long term cold storage.

## 2. Material and Methods

### 2.1. Chemicals

Lemongrass essential oil (LG) was purchased from Plena Natura (Portugal). Citral (95%) was purchased from Acrós-Organics (Madrid, Spain). Food grade sodium alginate and nonionic surfactant Tween80 (Polyoxyethylene sorbitan MonoOleate) were purchased from Sigma–Aldrich Chemic (Steinhein, Germany). Milli-Q filtration system (0.22 µm) was used for obtaining ultrapure water. Calcium chloride was from Sigma–Aldrich Chemic (Steinhein, Germany) and ascorbic acid from Scharlau (Barcelona, Spain). Tryptone Soy Broth (TSB), Tryptone Soy Agar (TSA), Plate Count Agar (PCA), Dicloran Rose-Bengal Chloramphenicol Agar (DRBC) and Potato Dextrose Agar (PDA) were from Bioakar Diagnostics (Beauvais, France) and Phosphate Buffered Saline Tablets and Glycerol from Fisher Scientific (Loughborough, UK).

### 2.2. Lemongrass Essential Oil (LG) Analysis

LG was analysed by gas chromatography (GC) and gas chromatography coupled to mass spectrometry (GC-MS), for compound quantification and identification, respectively, as described by Rodrigues et al. [16].

### 2.3. Preparation of Coating-Forming Nanoemulsions

Sodium alginate (2%, *w*/*w*) was dissolved in MilliQ water at 70 °C, with continuous stirring until complete dissolution, then the solution was cooled down to 25 °C [17]. The EOs were incorporated into the alginate solution (2%, *w*/*w*) using a thermomix (Vorwerk & Co.KG, Wuppertal, Germany) in 6 series of 1 min at speed 9 (3028 g), avoiding exceeding 37 °C. Thereafter, the emulsion was mixed with a T-18 Ultraturrax (IKA, Staufen, Germany) for 1 min at 1762 g. Concentrations of the LG and citral (Cit), used in the emulsions were based on their minimum inhibitory concentrations (MIC), respectively 0.25 and 0.20% (*w*/*w*) [17]. Nanoemulsion formulations were prepared using 5 or 10 times higher concentrations of LG and Cit, once they were applied to fruit with long-term storage, during which oil volatilization may occur. Tween 80 concentrations were bound at an oil/surfactant ratio of 1:3.

### 2.4. Nanoemulsion Characterization

Particle size, polydispersity and z-potential of nanoemulsions were determined by dynamic-light-scattering (DLS) and phase-analysis light scattering (PALS) according to Artiga-Artigas et al. [18]. To avoid multiple scattering effects, samples were diluted prior to analysis with Milli-Q water (1:10).

Nanoemulsions were observed by negative-staining electron microscopy as a direct measurement of their droplet size and shape as reported by Artiga-Artigas et al. [18]. The grids were observed in a transmission electron microscope Morgagni 268D TEM (FEI Company, Netherlands) with a CCD Mega-View camera (Olympus, Tokyo, Japan).

Cells THP-1 (human leukemia monocytic cell line) and HT-29 (human colon cancer cell line) were used to evaluate the cytotoxicity of coatings. Cell culture maintenance and cell viability were evaluated as described previously [4].

### 2.5. Fruit Coating

‘Rocha’ pear (*Pyrus communis* L.) were harvested at optimal storage ripening (Firmness 49.83 ± 2.24 N, SSC 12.97 ± 0.27 °Brix) [19] from orchards in the West Region of Portugal and immediately transported to the University of Algarve. Pears were dipped into nanoemulsion for 2 min, allowed to drip off for 1 min, then, dipped in calcium chloride 1% for 1 min. Treatments were sodium alginate 2% (*w*/*w*) + citral 1% (*w*/*w*) (Cit1%); sodium alginate 2% (*w*/*w*) + citral 2% (*w*/*w*) (Cit2%); sodium alginate 2% (*w*/*w*) + lemongrass 1.25% (*w*/*w*) (LG1.25%); sodium alginate 2% (*w*/*w*) + lemongrass2.5% (*w*/*w*) (LG2.5%) and uncoated fruit (control). The coatings formed on fruit were allowed to dry during 2 h at room temperature (25 °C), after that, fruit were placed in plastic crates and stored at 0 °C with relative humidity 95%. After 2, 4 and 6 months, three replicates per treatment, with 12 fruits per replicate were sampled for quality parameters, as well as after 7 d shelf-life at ~22 °C and 70% RH.

### 2.6. Quality Parameters

Fruit surface colour was measured with a Minolta Chroma meter CR-300 (EC Minolta, Japan) using the CIELab scale (L*, a*,b*). Hue was calculated as ◦Hue = arctan(b*/a*). Firmness was measured after skin removal with a Chatillon TCD200 and a Digital Force Gauge DFIS50 (Jonh Chatillon&Sons, Inc., Somerset, FL, USA) fitted with an 8 mm diameter probe [3]. Electrolyte leakage (EL) was assessed as described by Gago et al. [20]. The soluble solids content (SSC) were measured in the fruit juice by a digital refractometer PR1ATAGO CoLTD (Japan) and titrable acidity by titration with 0.1N NaOH. Weight loss was expressed as a percentage of the initial weight. Superficial scald and internal browning disorder (IBD) incidence were visually assessed as described in Gago et al. [3] and expressed as a percentage of the total fruit number.

### 2.7. Microbial Counts

The procedures for counts of aerobic mesophilic and psychrophilic bacteria and mould and yeasts were performed according to Guerreiro et al. [4]. Fruit homogenates were prepared by using a masticator homogenizator classic/panoramic (IUL Instruments, Barcelona, Spain).

### 2.8. Sensory Evaluation

A taste panel of 15 recruited people from academic staff and students who are familiar with those taste panels, was used to evaluate sensory parameters (fruit appearance, pulp appearance, aroma, texture, sweetness, acidity, overall flavour) on a 7-point hedonic scale (1-bad; 7-excellent) at the end of the experiment.

### 2.9. Statistical Analysis

The experimental design was a complete randomized block design. Statistical analysis was performed using the SPSS 24.0 software (IBM, Inc., Armonk, N.Y., USA). Two-way analysis of variance (ANOVA) was done using treatments and storage time as factors. Duncan’s multiple-range tests (*p* < 0.05) for means comparison were done.

## 3. Results and Discussion

### 3.1. Lemongrass Essential Oil (LG) Composition

Lemongrass chromatographic analysis identified twenty-one compounds, accounting for 97% of the total EO, which are listed in Table 1 in order of their elution on the DB-1 column. LG chemical profile was dominated by oxygen-containing monoterpenes (91%), neral (35%), geranial (34%) and geraniol (17%) being the main compounds. Geranial (23–45%), neral (20–36%), β-myrcene (traces–38%), and geraniol (1–18%) have been reported as the main compounds of this species’ EOs [21]. Despite the lower levels of β-myrcene, the main compounds of the essential oil assessed in this study were in agreement with these ranges (Table 1).

### 3.2. Nanoemulsion Characterization

#### 3.2.1. Droplet Size, Polydispersity and Zeta-Potential

Despite some differences in nanoemulsions’ droplet size (Table 2), all formulations investigated had droplets in the nano range, smaller than 500 nm [14]. However, in LG-nanoemulsion, the particles’ size was approximately twice the size of Cit-nanoemnulsions’ droplets. This finding can be attributed to the significant differences among zeta potential (Table 2), since lower negative zeta potential values present in LG -nanoemulsions may not be sufficient for creating an energy barrier between droplets that prevent coalescence [22]. Recently, Gago et al. [17] reported that nanoemulsions with the same composition, obtained from coarse emulsions submitted to microfluidization, presented similar droplets size for both nanoemulsions containing Cit and LG. Although not statistically significant at *p* > 0.05, there was a tendency for the nanoemulsions having higher EO concentration to contain smaller droplets.

Polydispersity values <0.3 mean good uniformity of particle size within the formulation [23]. The higher the polydispersity index, the lower the uniformity of the droplet size in the formulation. In this experiment, a higher concentration of LG and Cit showed uniformity of the particles’ size since they had a polydispersity index of 0.26 and 0.31, respectively (Table 2). The polydispersity index for the other nanoemulsions was 0.36, slightly higher. However, all these polydispersity values were almost half of those reported by Gago et al. [17] for the same nanoemulsion composition. This may be due to the nanoemulsions production process, which contributes to reach more uniform size droplets.

The amplitude of the electrostatic or charge repulsion/attraction between particles can be measured by the zeta potential [24]. Surface potential (zeta potential) formed by surfactants can produce repulsive/attractive electrical forces among approaching oil droplets and thus prevents their coalescence. The results of zeta potential measurements are presented in Table 2 and show a negative charge in the range of −52.84 to −23.10 mV, which corresponds to the stable dispersion of the droplets backed by non-ionic low-mass nature of Tween 80 [18]. Gago et al. [17] reported similar zeta potential values for Cit-nanoemulsions, but values were less negative for LG-nanoemulsions.

#### 3.2.2. Microstructure

**Figure 1:**
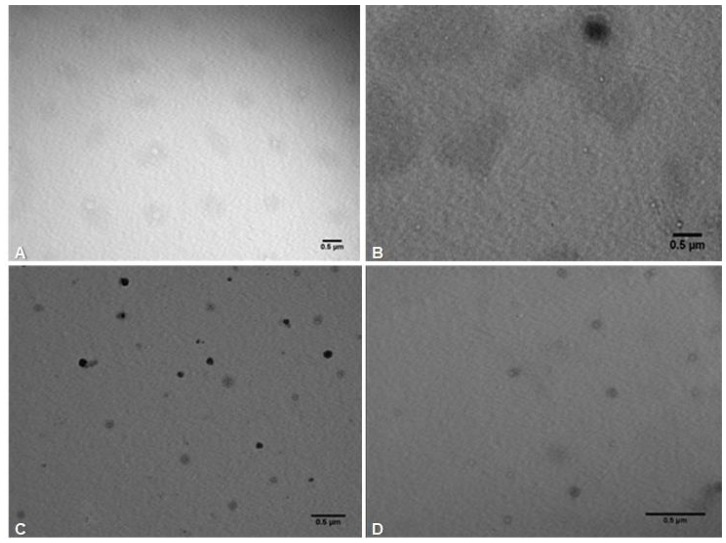
Transmission electron microscopy (TEM) images of nanoemulsions (**A**) (Cit1%), (**B**) (Cit2%), (**C**) (LG1.25%) and (**D**) (LG2.5%).

The morphology of droplets in Cit1%, Cit2%, LG1.25% and LG2.5% nanoemulsions was observed by TEM (Figure 1). TEM images showed an average droplet size of 111.8 ± 54.84 nm in Cit1% (Figure 1A) and 96.5 ± 19.4 nm in Cit2% (Figure 1B), while particles’ size calculated by DLS were lower, respectively, 42.15 ± 11.42 nm and 26.04 ± 5.43 nm. In the case of LG-nanoemulsions, the particle size obtained by TEM images were 87.96 ± 18.40 nm and 60.43 ± 17.34 nm, respectively, in LG1.25% (Figure 1C) and LG2.5% (Figure 1D), which in this case, were similar to those measured by DLS (82.81 ± 39.28 nm and 68.28 ± 21.84 nm) (Table 2). The differences among droplet sizes observed can be partly explained by the different principles for measuring particle sizes by DLS and TEM. In DLS, a dynamic method that is sensitive to the dispersion and/or aggregation of the nanoparticles in solution, the evaluation of nanoparticle or droplet sizes is based on the determination of the frequency of their movement, being highly dependent on the refractive index of the liquid sample. In TEM there is a direct measurement of droplet sizes [25]. Both methods provide important information: DLS reveals details about the dynamics and stability of the nanoparticles, whereas TEM gives details not only about the particle size but also about their shape [25].

#### 3.2.3. Nanoemulsions Cytotoxicity

**Figure 2:**
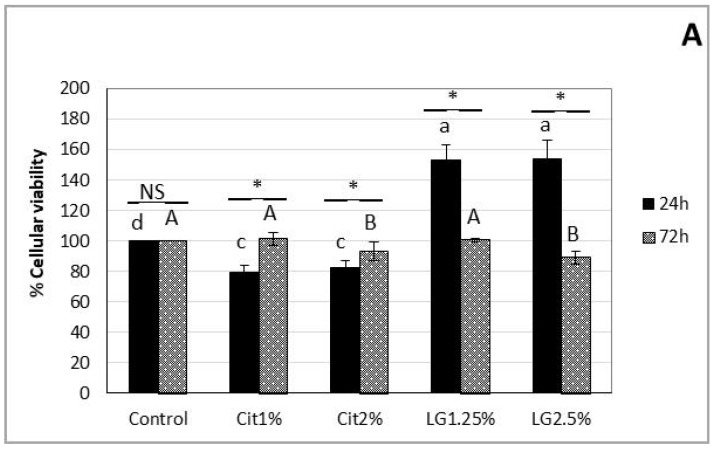

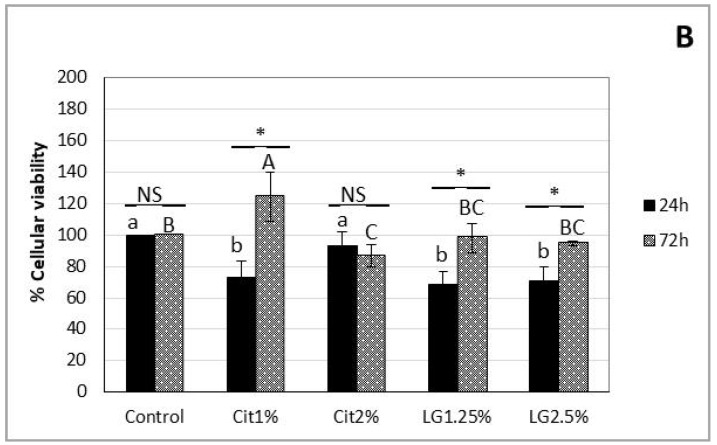
THP-1 (**A**) and HT-29 (**B**) cells percentage viability in the presence of Cit1%, Cit2%, LG1.25% and LG2.5% nanoemulsions and control (without nanoemulsions). The MTT assay was carried out after incubation for 24 h and 72 h. Data represent mean ± SD of five incubations. Bars with the same lower-case letter at 24 h and bars with the same upper case letter at 72 h are not significantly different by Duncan’s Multiple Range Test, at *p* < 0.05. Where the paired bars are marked NS or *, they are not significantly different or significant at *p* < 0.05, respectively, from each other.

The potential cytotoxic effect of the nanoemulsions tested in the current study was examined on THP-1 and HT-29 cell lines (Figure 2). After 24 h, the THP-1 cells exhibited significantly (*p* < 0.05) higher cell viability when treated with LG-nanoemulsions but significantly lower (*p* < 0.05) in the case of Cit-nanoemulsions in with the control (Figure 2A). However, after 72 h of exposure, the viability of THP-1 was similar (*p* > 0.05) to the control and all nanoemulsions tested. In the case of HT-29 cells, after 24 h of exposure to nanoemulsions, it was observed a significant reduction (*p* < 0.05) of the cell viability in all nanoemulsions tested, with the exception of Cit2%. However, after 72 h of exposure to nanoemulsions, the viability of HT-29 cells was similar to control for LG-nanoemulsions (Figure 2B), which suggests that formulations did not induce toxicity against the tested cell lines. Intriguing, the viability of HT-29 cells exposed to Cit1% increased after 72 h, being higher than the control (Figure 2B), evidencing the need for further investigation.

### 3.3. Fruit Quality Parameters

#### 3.3.1. Colour

Initially, right after the application of coatings on pears, the L* and *hue* values were not significantly different among the fruits covered with the different coatings and control, suggesting that coatings had no effect on pears’ colour (Table 3). In fact, the maintenance of food colour after the application of nanoemulsions is due to their optical clarity and is considered one of their advantages over conventional emulsions [26]. However, throughout the cold storage and shelf-life period, coatings slowed down the ripening process (Table 3).

During storage, there was a progressive increase in L* and a reduction in *hue* values in all fruit (Table 3), which characterize the ripening of ‘Rocha’ pears [3]. Nevertheless, after four months cold storage, the uncoated pears maintained the highest L* and the lowest *hue* values, indicating faster ripening. The same trends were maintained in the shelf-life periods. Such results indicate that nanocoatings have a greater effect on green colour retention, thus slowing the ripening process.

#### 3.3.2. Firmness

Firmness decreased in all fruits upon storage and shelf-life (Table 3). Upon removal from cold storage, uncoated pears showed significantly lower values than coated-fruit up to four months, while after six months storage, Cit were the fruit with the higher firmness values. After 7d shelf-life, the firmness values decreased more sharply in the control followed by fruits coated with Cit-nanoemulsions, mainly the higher concentration. The LG samples showed the highest firmness values after shelf-life through the experiment, with adequate and desirable firmness values for consumption (~24 N), while Cit-coated and, specifically, the uncoated fruits were too soft, close to overripe.

In the present work, the incorporation of CaCl_2_ for polymerisation of the coatings could be an important factor in preserving the firmness [12]. However, the differences among nanocoatings also show an additional effect with each coating. The moisture barrier property of the coating might, eventually, be reduced by the incorporation of EO, thus suffer a loss of firmness. This may be the case of Cit at higher concentrations which has been reported to reduce firmness in other fruit [5].

#### 3.3.3. SSC and TA

After coatings application, SSC values were between 12.23% and 12.97% and increased during storage in all treatments, mostly in the first two months. This may be due to starch turning into soluble sugars in fruit in earlier storage. After two months of cold storage, uncoated pears and those coated with LG-nanoemulsions had higher SSC, then until the end of the storage period, SSC values were similar in all fruit. Therefore, coatings had an effect on the SSC of ‘Rocha’ pears only at the beginning of the cold storage period. Medeiros et al. [27] reported a similar profile with polysaccharide/protein nanomultilayer coatings. Initially, in the shelf-life period that followed the coating application, there was a significant increase in the SSC values in all fruits. However, in the shelf-life periods after cold storage, all treatments maintained their SSC values, except Cit2% which decreased their SSC at the end of storage (Table 3).

During shelf-life after harvest and during the first two months of storage, TA decreased significantly in all treatments, as expected due to the ripening process (Table 3). Thereafter, a slow decrease was observed in all fruit, as reported by Galvis-Sánchez et al. [28].

#### 3.3.4. Electrolyte Leakage (EL)

EL values were similar in all pears just after treatment application (Table 3). During the first two months of cold storage, EL decreased in all pears, probably due to structural changes and/or membrane compounds reorganization due to cold acclimatization [29]. After four months of cold storage, EL was higher in control followed by Cit and the lower values were in LG-coated fruit. This difference seems to reflect higher permeability of the membrane in the uncoated fruit during cold storage. This effect was more evident after their respective shelf-life, which confirms the possibility of the Cit to damage cells, thus inducing firmness loss as reported above [5].

Reduced electrolytic leakage due to edible coatings application was reported for mango [30] and jujube fruits [31], with chitosan coatings and chitosan with cinnamon oil, respectively. Those results were attributed to the chitosan protective effect and also to the antioxidant activity of cinnamon oil.

#### 3.3.5. Weight Loss

An increase in weight loss was noticed in all fruits upon storage and shelf-life (Table 3). Control and coated fruit had similar weight loss through storage, being slightly higher in LG-coated fruit. Nevertheless, after six months of respective shelf-life, weight loss was similar for all treatments. Medeiros et al. [27] found reduced weight loss in ‘Rocha’ pears coated with polysaccharide/protein nanomultilayer. However, Rojas-Graü et al. [12] found no differences in fresh cut ‘Fuji’ apples coated with alginate.

#### 3.3.6. Aerobic Mesophilic Microorganisms and Yeasts and Moulds

Our experiment showed very low yeasts and mould growth, showing values from 0.00 to 0.43 Log CFU/g even after six months storage plus 7 d shelf-life (Table 4). Some EOs are reported to have antimicrobial action [17]. Additionally, the barrier created by the edible coating protects food from microbial spoilage [12]. Adding EOs to edible coatings improves its antimicrobial action [4,5]. However, in the present study, such benefit was not evident due to the lack of microbial spoilage. Similar results were found for aerobic mesophilic microorganisms (Table 4).

According to Bierhals et al. [32], the limit of acceptance for the consumption of fruit products is 6 Log CFU/g, our counts of yeasts and mould or mesophilic aerobic microorganisms did not reach that limit during all the experiment. No growth of psychrophilic aerobic bacteria was found.

#### 3.3.7. Superficial Scald and Internal Browning

**Figure 3:**
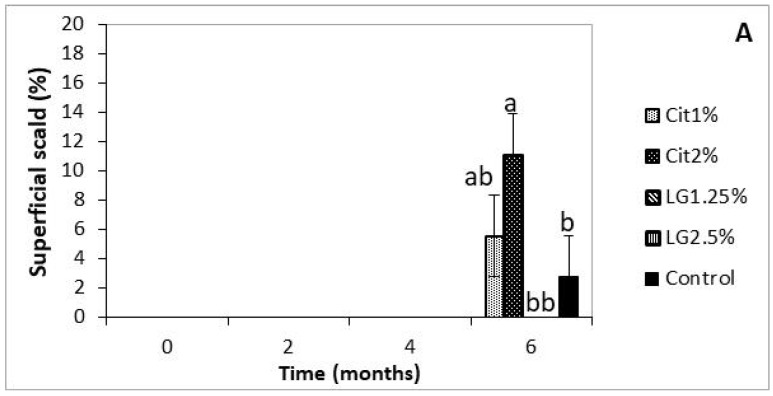

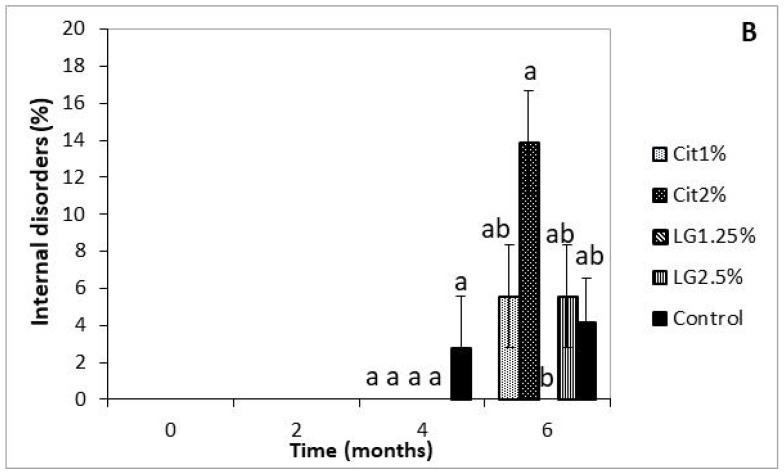
Effect of nanocoatings on superficial scald (**A**) and internal disorders (**B**) of ‘Rocha’ pears, after 0, 2, 4 and 6 months storage at 0 °C plus 7 d shelf-life at ≈22 °C. Uncoated control, Cit1%, Cit2%, LG1.25% and LG2.5% nanoemulsions. Bars in the same time (months) followed by the same case letter are not significant different at *p* < 0.05.

Chilling induced physiological disorders appeared only at the end of the storage period, with the exception of internal browning which appeared first for control fruit after four months storage plus 7 d shelf-life (Figure 3A,B).

For both, superficial scald and internal browning, fruit coated with the higher concentration of Cit2%, showed the highest percentage of affected fruit, while fruit coated with the lower concentration of LG1.25% showed none of the disorders. Moreover, LG-nanoemulsions did not show superficial scald, and the other treatments had intermediate values (Figure 3A).

It seems that Cit and LG-nanoemulsions have opposite effects on the development and appearance of the superficial scald in ‘Rocha’ pears, respectively, promoting and avoiding it. The larger size of LG-nanoemulsions droplets compared to Cit-nanoemulsions or the composition of the LG seems to avoid superficial scald. Thus, it appears that one or more of the compounds which are present in the LG composition rather than Cit may be responsible for this protective effect. It is known that 6-Methylhept-5-en-2-one (MHO), also present in LG composition (Table 1), has been associated to scald development [2]. According to Pesis et al. [33], the conversion of MHO-one to 6-methyl-5-hepten-2-ol (MHO-ol) in low O_2_-stress ‘Granny Smith’ apples is responsible for the lower scald development.

The application of edible coatings can create inside fruits a modified atmosphere due to resistance to gas diffusion leading to a reduction of the senescence process [12,27,34]. In fact, alginate edible coatings have been reported to reduce gas exchange [6]. Given all this, it is possible to hypothesize that the LG-coated pears were able to convert the existing MHO-one to MHO-ol, which allowed the fruit to be more protected from superficial scald development, which needs further investigation.

Uncoated fruits were the first showing IBD, after four months of storage. For internal browning, increasing concentrations of essential oils showed increased disorder (Figure 3B). As for scald, LG1.25% coated fruit did not show symptoms of IBD.

It is known that several coatings can reduce gas transfer rates in many studied fruits [34]. Although low oxygen storage is beneficial to reduce superficial scald, too low O_2_ level combined with high CO_2_ leads to the development of IBD [1]. Interestingly, coatings with a higher concentration of Cit (neral and geranial) are the ones with higher internal browning, suggesting an effect of these isomers on the development of the disorder. The effect of LG on reducing the main chilling induced physiological disorders in pears seems promising and needs further research.

#### 3.3.8. Sensory Evaluation

EOs-based nanoemulsions have been applied in food products to extend their shelf-life by keeping or improving their appearance, flavour, aroma as well as nutritional quality [13,35]. However, they can change their sensory properties.

**Figure 4:**
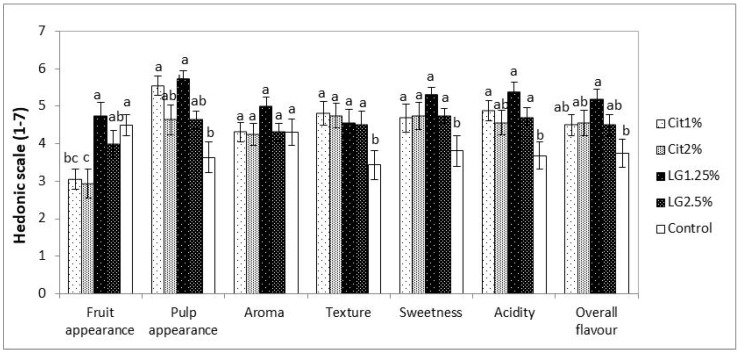
Sensory evaluation of ‘Rocha’ pears coated with different nanoemulsions (Cit1%, Cit2%, LG1.25% and LG2.5%) and control (uncoated fruits) after 6 months of cold storage at 0 °C plus 7 days of shelf-life. Bars with the same lower case letter in day 0, and bars with the same upper case letter in the same parameter are not significantly different by Duncan’s Multiple Range Test, at *p* < 0.05.

In our work, after six months cold storage plus 7 d shelf-life, appearance, which is the first attribute for consumers’ decision to buy, was over a good sensory appreciation (≥4.0 in a scale of 1-dislike definitely to 7-like definitely) for control and LG coated fruit, while Cit-coated were ranked negatively (around 3) (Figure 4). Nevertheless, while evaluating texture, sweetness and acidity, controls performed lower than all coated fruit.

According to taste panels, pears scored higher in overall liking in LG1.25%-coated fruits and the less appreciated in texture, sweetness and acidity were uncoated pears (Figure 4). Interestingly, the aroma was not affected by the coatings, with the LG1.25% being slightly better appreciated than the other treatments.

## 4. Conclusions

The present research has established the proof-of-concept of the use of edible coatings for improving postharvest quality in long-term cold storage life of ’Rocha´ pear. The tested coatings were composed of droplets in the nano range dimension with no effect on pear initial colour. Their effect was on slowing down the colour evolution during storage and shelf-life by reducing the ripening process. Additionally, uncoated pears presented lower firmness and higher electrolytic leakage when compared to coated pears proving their effect on retarding the ripening process. In addition, pears coated with LG nanoemulsions had no superficial scald symptoms and the edible coatings LG1.25% did not show IBD and was effective in keeping sensory quality for up to six months plus 7 d shelf-life. These findings suggest that LG nanoemulsions, mainly LG1.25%, used in pears coatings, had a significant positive effect on the quality of the fruit and, therefore, hold potential for preventing the superficial scald and IBD in long term cold storage. The effect of LG at the lower concentration seems promising for reducing chilling induced disorders, thus needs further investigation.

## Figures and Tables

**Table 1:** Percentage composition of lemongrass (*Cymbopogon citratus*) essential oil.

Compounds	RI	%
Tricyclene	921	0.4
α-Fenchene	938	t
Camphene	938	1.7
6-Methylhept-5-en-2-one	960	1.0
β-Myrcene	975	0.3
1,8-Cineole	1005	0.4
Limonene	1009	0.4
Linalool	1074	1.1
Citronellal	1121	0.4
α-Terpineol	1159	0.3
Neral	1210	34.8
Piperitone	1211	0.1
Geraniol	1236	16.9
Geranial	1240	33.7
Geranyl acetate	1370	3.2
β-Caryophyllene	1414	1.2
*trans*-α-Bergamotene	1434	t
α-Humulene	1447	0.1
δ-Cadinene	1505	0.1
Elemol	1530	0.3
β-Caryophyllene oxide	1561	0.4
% of Identification		96.8
Grouped compounds		
Monoterpene hydrocarbons		2.8
Oxygen-containing monoterpenes		90.9
Sesquiterpene hydrocarbons		1.4
Oxygen-containing sesquiterpenes		0.7
Others		1.0

RI: In-lab calculated Retention Index relative to C_9_-C_16_ *n*-alkanes on the DB-1column, t: trace (*p* < 0.05%).

**Table 2:** Droplet size (nm), polydispersity index (PDI) and zeta-potential (mV) of nanoemulsions containing sodium alginate and lemongrass essential oil and its main compound citral.

Nanoemulsion	Droplet Size (nm)	Polydispersity Index	Zeta-Potential (mV)
Cit1%	42.15 ± 11.42 ^bc^	0.36 ± 0.03 ^a^	−52.84 ± 3.18 ^c^
Cit2%	26.04 ± 5.43 ^c^	0.31 ± 0.04 ^ab^	−37.79 ± 8.53 ^b^
LG1.25%	82.81 ± 39.28 ^a^	0.36 ± 0.04 ^a^	−28.29 ± 10.69 ^ab^
LG2.5%	68.28 ± 21.84 ^ab^	0.26 ± 0.05 ^b^	−23.10 ± 6.53 ^a^

^a–c^ Means in same column with different letters are significantly different at *p* < 0.05. Data shown are the means ± SD.

**Table 3:** Changes in colour parameters (L*, Hue), firmness (Fm), soluble solid content (SSC), titratable acidity (TA), electrolytic leakage (EL) and weight loss during storage (0, 2, 4 and 6 months) and post storage shelf-life (7 days) of uncoated (control) and coated ‘Rocha’ pears with nanoemulsions Cit1%, Cit2%, LG1.25% and LG2.5%.

Quality Parameters	0 Month		2 Months		4 Months		6 Months	
Nanoemulsion	0 d	7 d	0 d	7 d	0 d	7 d	0 d	7 d
Lightness (L*)								
Cit1%	71.02 ± 0.52 ^aD^	71.17 ± 0.49 ^aD^	74.03 ± 0.49 ^cC^	74.91 ± 0.45 ^bBC^	75.45 ± 0.50 ^cB^	75.56 ± 0.62 ^bB^	77.31 ± 0.35 ^aA^	77.87 ± 0.21 ^abA^
Cit2%	70.36 ± 0.37 ^aC^	70.42 ± 0.41 ^abDC^	73.61 ±.62 ^cB^	73.76 ± 0.55 ^bcB^	76.98 ± 0.25 ^bcA^	77.43 ± 1.21 ^abA^	78.04 ± 0.27 ^aA^	77.04 ± 0.31 ^bA^
LG1.25%	71.16 ± 0.39 ^aD^	70.30 ± 0.42 ^abD^	75.64 ± 0.29 ^bB^	73.13 ± 0.61 ^cdC^	76.10 ± 0.55 ^bcB^	76.01 ± 0.56 ^abB^	78.04 ± 0.31 ^aA^	77.71 ± 0.37 ^abA^
LG2.5%	70.80 ± 0.45 ^aCD^	69.41 ± 0.57 ^bD^	75.06 ± 0.62 ^bcB^	72.08 ± 0.54 ^dC^	76.50 ± 0.65 ^bcA^	76.76 ± 0.34 ^abA^	77.79 ± 0.32 ^aA^	77.76 ± 0.37 ^abA^
Control	71.39 ± 0.58 ^aB^	71.45 ± 0.49 ^aB^	77.30 ± 0.39 ^aA^	78.20 ± 0.39 ^aA^	78.02 ± 0.30 ^aA^	77.90 ± 0.29 ^aA^	77.62 ± 0.25 ^aA^	78.02 ± 0.25 ^aA^
Hue angle (*h*)								
Cit1%	108.86 ± 0.71 ^aA^	107.33 ± 0.61 ^bcA^	105.44 ± 0.53 ^aB^	103.04 ± 0.80 ^aC^	100.35 ± 0.62 ^aD^	95.85 ± 0.73 ^aE^	93.77 ± 0.53 ^aF^	94.42 ± 0.31 ^aEF^
Cit2%	109.96 ± 0.53 ^aA^	109.43 ± 0.49 ^aA^	105.14 ± 0.63 ^abB^	103.05 ± 0.63 ^aC^	98.30 ± 0.44 ^bD^	96.18 ± 0.61 ^aE^	94.08 ± 0.33 ^abF^	92.79 ± 0.36 ^bF^
LG1.25%	109.59 ± 0.53 ^aA^	108.87 ± 0.44 ^abA^	103.54 ± 0.49 ^bcB^	104.02 ± 0.62 ^aB^	97.98 ± 0.55 ^bC^	97.00 ± 0.56 ^aC^	93.10 ± 0.47 ^abD^	93.42 ± 0.37 ^bD^
LG2.5%	109.83 ± 0.49 ^aA^	108.91 ± 0.58 ^abA^	102.73 ± 0.58 ^cC^	104.31 ± 0.39 ^aB^	96.37 ± 0.49 ^cD^	95.79 ± 0.36 ^aD^	91.66 ± 0.60 ^cE^	92.52 ± 0.29 ^bE^
Control	108.76 ± 0.70 ^aA^	106.20 ± 0.99 ^cB^	98.35 ± 0.69 ^dC^	93.21 ± 0.36 ^bDE^	94.70 ± 0.28 ^dD^	91.48 ± 0.31 ^bE^	92.36 ± 0.36 ^bcE^	91.59 ± 0.24 ^cE^
Firmness (N)								
Cit1%	52.01 ± 1.98 ^aA^	39.83 ± 2.93 ^abC^	45.98 ± 2.45 ^bABC^	19.18 ± 3.55 ^abD^	48.22 ± 1.32 ^aAB^	14.95 ± 3.34 ^bcD^	43.74 ± 1.16 ^abBC^	19.18 ± 2.22 ^abD^
Cit2%	51.19 ± 1.88 ^aA^	45.37 ± 2.25 ^aB^	51.68 ± 1.39 ^aA^	15.45 ± 3.18 ^bcC^	46.35 ± 0.91 ^abAB^	14.96 ± 2.41 ^bcC^	44.26 ± 1.35 ^aB^	14.42 ± 1.45 ^bcC^
LG1.25%	47.34 ± 1.32 ^aA^	40.89 ± 2.56 ^abA^	46.04 ± 0.97 ^bA^	22.54 ± 3.31 ^abB^	45.28 ± 1.64 ^abA^	23.72 ± 3.20 ^aB^	40.73 ± 1.05 ^bcA^	21.01 ± 1.78 ^aB^
LG2.5%	48.98 ± 1.77 ^aA^	37.57 ± 3.53 ^abC^	45.30 ± 1.65 ^bAB^	26.94 ± 3.64 ^aD^	43.69 ± 0.87 ^bcABC^	21.13 ± 2.22 ^abD^	39.44 ± 1.15 ^cBC^	20.41 ± 2.75 ^aD^
Control	49.83 ± 2.24 ^aA^	33.47 ± 4.79 ^bC^	39.95 ± 2.45 ^cB^	7.63 ± 0.50 ^cD^	40.68 ± 1.21 ^cB^	7.78 ± 0.81 ^cD^	40.73 ± 0.91 ^bcB^	9.63 ± 0.53 ^cD^
SSC (%)								
Cit1%	12.63 ± 0.09 ^abB^	13.63 ± 0.28 ^bcA^	13.43 ± 0.19 ^bAB^	14.10 ± 0.21 ^aA^	13.93 ± 0.12 ^aA^	14.07 ± 0.29 ^aA^	13.87 ± 0.17 ^aA^	13.97 ± 0.21 ^aA^
Cit2%	12.23 ± 0.07 ^bD^	13.17 ± 0.29 ^cBC^	13.43 ± 0.09 ^bBC^	13.83 ± 0.09 ^aAB^	13.43 ± 0.09 ^aBC^	13.87 ± 0.24 ^aAB^	14.43 ± 0.38 ^aA^	12.97 ± 0.15 ^bC^
LG1.25%	12.33 ± 0.19 ^bB^	13.87 ± 0.18 ^bcA^	14.07 ± 0. 07 ^aA^	14.40 ± 0.35 ^aA^	14.30 ± 0.25 ^aA^	14.47 ± 0.21 ^aA^	14.30 ± 0.28 ^aA^	13.80 ± 0.29 ^abA^
LG2.5%	12.63 ± 0.23 ^abB^	14.17 ± 0.17 ^abA^	14.30 ± 0.15 ^aA^	14.10 ± 0.26 ^aA^	14.10 ± 0.37 ^aA^	14.40 ± 0.26 ^aA^	14.40 ± 0.21 ^aA^	13.63 ± 0.35 ^abA^
Control	12.97 ± 0.27 ^aC^	14.70 ± 0.3 ^aA^	14.13 ± 0.17 ^aAB^	13.80 ± 0.10 ^aABC^	14.20 ± 0.41 ^aAB^	13.70 ± 0.25 ^aBC^	14.03 ± 0.32 ^aAB^	13.27 ± 0.32 ^abBC^
Titratable acidity (g.mL^−1^ malic acid)								
Cit1%	0.42 ± 0.03 ^aA^	0.18 ± 0.01 ^abB^	0.16 ± 0.00 ^aB^	0.16 ± 0.00 ^abB^	0.13 ± 0.01 ^aC^	0.10 ± 0.00 ^abC^	0.10 ± 0.01 ^cC^	0.11 ± 0.00 ^aC^
Cit2%	0.43 ± 0.06 ^aA^	0.20 ± 0.01 ^aB^	0.15 ± 0.01 ^abBC^	0.18 ± 0.01 ^aBC^	0.11 ± 0.01 ^aC^	0.11 ± 0.00 ^abC^	0.15 ± 0.01 ^aBC^	0.12 ± 0.01 ^aC^
LG1.25%	0.39 ± 0.00 ^aA^	0.15 ± 0.01 ^bBC^	0.14 ± 0.01 ^bBC^	0.16 ± 0.01 ^abB^	0.12 ± 0.01 ^aD^	0.13 ± 0.00 ^aCD^	0.14 ± 0.01 ^abBCD^	0.12 ± 0.00 ^aD^
LG2.5%	0.45 ± 0.03 ^aA^	0.19 ± 0.01 ^aB^	0.15 ± 0.01 ^abC^	0.14 ± 0.00 ^bC^	0.12 ± 0.01 ^aC^	0.12 ± 0.01 ^aC^	0.12 ± 0.02 ^abcC^	0.13 ± 0.00 ^aC^
Control	0.49 ± 0.02 ^aA^	0.17 ± 0.01 ^abcB^	0.14 ± 0.00 ^bBC^	0.16 ± 0.01 ^abBC^	0.14 ± 0.01 ^aBC^	0.09 ± 0.02 ^bD^	0.11 ± 0.00 ^bcCD^	0.11 ± 0.00 ^aCD^
Electrolytic leakage (%)								
Cit1%	54.23 ± 0.79 ^aC^	58.15 ± 0.80 ^aC^	42.41 ± 1.39 ^bE^	69.40 ± 4.02 ^aB^	46.36 ± 1.95 ^bDE^	69.59 ± 3.63 ^bB^	52.72 ± 0.77 ^bCD^	76.49 ± 0.34 ^bA^
Cit2%	49.12 ± 1.41 ^aC^	59.60 ± 1.77 ^aB^	41.82 ± 1.02 ^bC^	69.62 ± 2.65 ^aA^	45.35 ± 1.21 ^bC^	68.60 ± 1.58 ^bA^	48.10 ± 2.67 ^bcC^	75.37 ± 6.74 ^bA^
LG1.25%	49.66 ± 1.24 ^aCD^	56.07 ± 2.17 ^aBC^	41.92 ± 1.30 ^bE^	61.02 ± 3.32 ^abB^	43.2 ± 1.34 ^bDE^	54.89 ± 0.82 ^cBC^	43.51 ± 1.83 ^cDE^	71.39 ± 3.04 ^bA^
LG2.5%	51.44 ± 2.17 ^aBC^	55.49 ± 0.34 ^aBC^	40.38 ± 3.02 ^bE^	58.92 ± 1.21 ^bB^	42.39 ± 1.07 ^bDE^	53.85 ± 4.38 ^cBC^	48.36 ± 2.09 ^bcCDE^	71.33 ± 2.03 ^bA^
Control	50.56 ± 2.62 ^aC^	60.12 ± 3.76 ^aB^	49.43 ± 3.25 ^aC^	62.60 ± 3.04 ^abB^	55.09 ± 2.10 ^aBC^	83.04 ± 1.51 ^aA^	59.83 ± 1.61 ^aB^	88.26 ± 1.88 ^aA^
Weight loss (%)								
Cit1%	0.00 ± 0.00 ^aF^	1.66 ± 0.03 ^abE^	2.08 ± 0.05 ^cE^	3.63 ± 0.05 ^cD^	4.34 ± 0.29 ^bC^	6.66 ± 0.24 ^cB^	6.57 ± 0.33 ^bB^	7.64 ± 0.39 ^bA^
Cit2%	0.00 ± 0.00 ^aH^	1.65 ± 0.03 ^abG^	2.23 ± 0.03 ^cF^	3.96 ± 0.03 ^bE^	4.40 ± 0.06 ^bD^	6.99 ± 0.18 ^bcB^	6.63 ± 0.05 ^bC^	7.84 ± 0.02 ^abA^
LG1.25%	0.00 ± 0.00 ^aG^	1.70 ± 0.05 ^aF^	2.49 ± 0.06 ^bE^	4.13 ± 0.06 ^bD^	5.00 ± 0.17 ^aB^	7.42 ± 0.20 ^abB^	7.36 ± 0.25 ^aB^	8.45 ± 0.28 ^abA^
LG2.5%	0.00 ± 0.00 ^aG^	1.80 ± 0.09 ^aF^	2.90 ± 0.08 ^aE^	4.44 ± 0.17 ^aD^	5.54 ± 0.20 ^aC^	7.93 ± 0.21 ^aB^	7.53 ± 0.19 ^aB^	8.59 ± 0.22 ^aA^
Control	0.00 ± 0.00 ^aG^	1.51 ± 0.04 ^bF^	2.08 ± 0.03 ^cE^	3.42 ± 0.02 ^cD^	4.25 ± 0.07 ^bC^	6.56 ± 0.07 ^cB^	6.72 ± 0.08 bB	7.8 ± 0.11 ^abA^

Values are means ± SD; The values followed by the same lower-case letter, in the same column and parameter and by the same upper-case letter in the same row are not significantly different (Duncan’s new multiple range test at *p* < 0.05).

**Table 4:** Effect of nanoemulsions Cit1%, Cit2%, LG1.25% and LG2.5% coatings in ‘Rocha’ pears on aerobic mesophilic microorganisms and yeast and moulds development during storage (0, 2, 4 and 6 months) and post storage shelf-life (7 days).

Microorganisms	0 Months		2 Months		4 Months		6 Months	
Nanoemulsion	0 d	7 d	0 d	7 d	0 d	7 d	0 d	7 d
Yeast and moulds (Log CFU/g)								
Cit 1%	0.67 ± 0.33 ^aA^	0.33 ± 0.33 ^aA^	0.67 ± 0.33 ^aA^	0.67 ± 0.33 ^abA^	0.93 ± 0.47 ^abA^	1.00 ± 0.00 ^aA^	0.41 ± 0.22 ^aA^	0.33 ± 0.33 ^aA^
Cit 2%	0.77 ± 0.39 ^aA^	0.33 ± 0.33 ^aA^	0.77 ± 0.39 ^aA^	0.33 ± 0.33 ^bA^	0.33 ± 0.33 ^bA^	0.00 ± 0.00 ^bA^	0.17 ± 0.17 ^aAB^	0.00 ± 0.00 ^aA^
LG 1.25 %	1.00 ± 0.00 ^aA^	0.00 ± 0.00 ^aB^	1.00 ± 0.00 ^aA^	0.00 ± 0.00 ^bB^	0.67 ± 0.33 ^bAB^	0.77 ± 0.39 ^aAB^	0.38 ± 0.20 ^aBC^	0.43 ± 0.43 ^aAB^
LG 2.5 %	0.93 ± 0.47 ^aA^	0.00 ± 0.00 ^aB^	1.10 ± 0.10 ^aA^	0.00 ± 0.00 ^bB^	0.00 ± 0.00 ^bB^	0.00 ± 0.00 ^bB^	0.33 ± 0.17 ^aAB^	0.00 ± 0.00 ^aB^
Control	1.10 ±0.10 ^aBC^	0.83 ± 0.44 ^aBC^	1.20 ± 0.10 ^aB^	1.10 ± 0.10 ^aBC^	1.77 ± 0.04 ^aA^	1.26 ± 0.14 ^aAB^	0.58 ± 0.08 ^aD^	0.00 ± 0.00 ^aD^
Aerobic mesophilic Microorganisms (Log CFU/g)								
Cit 1%	3.72 ± 0.03 ^aA^	2.67 ± 0.11 ^bB^	1.32 ± 0.02 ^cC^	2.54 ± 0.16 ^aB^	1.72 ± 0.14 ^bcDE^	3.52 ± 0.09 ^aA^	2.44 ± 0.61 ^aB^	3.51 ± 0.04 ^aA^
Cit 2%	3.59 ± 0.02 ^aAB^	3.87 ± 0.02 ^aA^	2.38 ± 0.52 ^bCD^	1.19 ± 0.27 ^bE^	1.59 ± 0.19 ^cDE^	2.81 ± 0.05 ^cBC^	3.18 ± 0.60 ^aABC^	3.30 ± 0.17 ^aABC^
LG 1.25%	3.60 ± 0.06 ^aA^	0.71 ± 0.03 ^dF^	1.32 ± 0.09 ^cEF^	2.71 ± 0.07 ^aBC^	2.30 ± 0.20 ^abCD^	3.74 ± 0.06 ^aA^	3.26 ± 0.14 ^aAB^	1.94 ± 0.51 ^bDE^
LG 2.5%	3.44 ± 0.06 ^bAB^	0.68 ± 0.03 ^dE^	1.18 ± 0.09 ^cDE^	2.54 ± 0.02 ^aBC^	2.04 ± 0.28 ^abcCD^	3.54 ± 0.02 ^bA^	2.65 ± 0.51 ^aABC^	2.70 ± 0.56 ^abABC^
Control	2.85 ± 0.05 ^cC^	2.08 ± 0.06 ^cE^	3.69 ± 0.05 aA	2.50 ± 0.02 ^aD^	2.45 ± 0.11 ^aD^	3.50 ± 0.02 ^bB^	3.65 ± 0.00 ^aAB^	3.00 ± 0.03 ^abC^

Values are means ± SD; The values followed by the same lower-case letter, in the same column and parameter and by the same upper-case letter in the same row are not significantly different (Duncan’s new multiple range test at *p* < 0.05).
